# 115 Preliminary results of the current MSFiT-study: a controlled trial examining the potential effects of regular online exercise training in people with Multiple Sclerosis

**DOI:** 10.1093/eurpub/ckae114.102

**Published:** 2024-09-26

**Authors:** Charlotte Klump, Stephanie Woschek, Laura Anhorn, Herbert Temmes, Juliane Lamprecht

**Affiliations:** Institute for Health and Nursing Sciences Medical Faculty, Martin Luther University, Halle-Wittenberg, Germany; German Multiple Sclerosis Society, Hanover, Germany; Sherpa B.V., Nijmegen, Netherlands; German Multiple Sclerosis Society, Hanover, Germany; Institute for Health and Nursing Sciences Medical Faculty, Martin Luther University, Halle-Wittenberg, Germany

## Abstract

**Purpose:**

Physical exercise has both preventive and rehabilitative effects in people with Multiple Sclerosis (pwMS). However, there is still a lack of evidence in effects of online-exercise interventions. The MSFiT-study, a cooperation of German MS Society, University of Halle-Wittenberg (Germany) and Sherpa B.V. (Netherlands), aims at examining effects of weekly 45 minutes „DMSG functional training “(FT) online in pwMS. The intervention was concepted in 2019 as therapeutic group training and can currently only prescribed as face-to-face-training.

**Methods:**

Inclusion criteria were a confirmed MS-diagnosis, age ≥18, no experience in FT, no relapse in the past 4 weeks. Participants were free to choose between online or face-to-face training (FACE). Online-participants were randomly divided in online-intervention (ONLINE) and wait-list-control-group (WAIT). Participants of the FACE-group were not randomised due to low application. Before (T0) and after 3 months training (T1) all participants filled out a standardised questionnaire via the app MS sherpa or paper form: Inter alia, outcomes are walking ability (2MWT), cognitive function (SDMT), function of the upper extremities (DASH) and fatigue (FSMC). End of study is 31st of March 2024.

**Results:**

65 pwMS were recruited. Until now n = 47 pwMS completed T0, T1 (ON = 22, WAIT = 19, FACE = 6) and showed an adherence of at minimum 50%. Preliminary descriptive data (T0) show: pwMS in ONLINE-group (age = 45.9±9.19 years; disease duration = 10.17±7.74 years) had a patient-reported disability (PDDS) score of 2.09 (±1.89), a fatigue of 70.5±15.5 and a DASH score of 26.1±18.3. WAIT-group (age = 50.84±9.65 years; disease duration = 16.16±11.21 years) had a PDDS of 2.79 (±2.27), a fatigue score of 66.2±17.7 and a DASH of 26.4±17.2. FACE-group (age = 58.14±8.47 years; disease duration = 19.71±18.88 years) had a PDDS of 4.67 (±2.45), fatigue score of 73,8±16.7 and DASH of 42.9±28.7.

**Conclusions:**

Further analyses will be necessary once the study period ends. Most participants seem to be physically limited due to PDDS and fatigue score. Research findings will be a further step to bring more trust in online-options for people with disabilities who are not able to participate in trainings due to infrastructure, symptom burdens or other obligations. Furthermore, results should demonstrate the equivalence of the two group forms as a basis for health policy decisions.

